# Loop-mediated isothermal amplification (LAMP) test in the detection of uncomplicated malaria in pregnancy: a meta-analysis of diagnostic accuracy

**DOI:** 10.1186/s12936-022-04419-9

**Published:** 2022-12-22

**Authors:** Joseph Lee Teck Yon, Norah Htet Htet, Cho Naing, Wong Siew Tung, Htar Htar Aung, Joon Wah Mak

**Affiliations:** 1 School of Medicine, International Medical University, Kuala Lumpur, Malaysia; 2 University of Edinburgh, Old College, Edinburgh, EH8 9YL UK; 3 Division of Tropical Health and Medicine, James Cook University, Townsville, QLD Australia; 4 Institute of Research, Development and Innovation (IRDI), International Medical University, Kuala Lumpur, Malaysia

## Abstract

**Background:**

Due to relatively low malaria parasitaemia in pregnancy, an appropriate field test that can adequately detect infections in pregnant women presenting with illness or for malaria screening during antenatal care is crucially important. The objective was to evaluate the diagnostic accuracy of loop-mediated isothermal amplification **(**LAMP) for the detection of uncomplicated malaria in pregnancy.

**Methods:**

This was a meta-analysis of diagnostic accuracy. Relevant studies that assessed the diagnostic performance of LAMP for the detection of malaria in pregnancy were searched in health-related electronic databases including PubMed, Ovid, and Google Scholar. The methodological quality of the studies included was evaluated using the QUADAS-2 tool.

**Results:**

Of the 372 studies identified, eight studies involving 2999 pregnant women in five endemic countries that assessed the accuracy of LAMP were identified. With three types of PCR as reference tests, the pooled sensitivity of LAMP was 91% (95%CI 67–98%) and pooled specificity was 99% (95%CI 83–100%, 4 studies), and the negative likelihood ratio was 9% (2–40%). Caution is needed in the interpretation as there was substantial between-study heterogeneity (*I*^2^: 80%), and a low probability that a person without infection is tested negative. With microscopy as a reference, the pooled sensitivity of LAMP was 95% (95%CI 26–100%) and pooled specificity was 100% (95%CI 94–100%, 4 studies). There was a wide range of sensitivity and substantial between-study heterogeneity (*I*^*2*^: 83.5–98.4%). To investigate the source of heterogeneity, a meta-regression analysis was performed with covariates. Of these potential confounding factors, reference test (p: 0.03) and study design (p:0.03) had affected the diagnostic accuracy of LAMP in malaria in pregnancy. Overall, there was a low certainty of the evidence in accuracy estimates.

**Conclusion:**

The findings suggest that LAMP is more sensitive than traditional tests used at facilities, but the utility of detecting and treating these low-density infections is not well understood. Due to the limited number of studies with bias in their methodological quality, variation in the study design, and different types of reference tests further research is likely to change the estimate. Well-conceived large prospective studies with blinding of the index test results are recommenced.

**Supplementary Information:**

The online version contains supplementary material available at 10.1186/s12936-022-04419-9.

## Background

Malaria remains globally important in certain endemic populations, even with considerable progress in control since 2000. The World Health Organization (WHO) global technical strategy (GTS) for malaria 2016–2030 is to have a world free of malaria [1]. It is estimated that at least 25% of pregnant women are infected with malaria contributing to more than 20% of all maternal deaths [2, 3]. To eliminate malaria and prevent reintroduction, the capacity to detect infections is critical [4, 5].

Malaria infection during pregnancy has negative impacts on the mother, the fetus, and subsequent neonatal and infant development [1, 2]. For instance, pregnant women infected with malaria, especially *Plasmodium falciparum* have a higher risk of developing maternal anaemia, maternal death, miscarriage, stillbirth and neonatal death [1, 2]. Moreover, parasite densities are often low and parasite sequestration may play a role in the fact that many malaria infections in pregnancy fall below the level of detection of light microscopy and rapid diagnostic test [6]. The drugs recommended for intermittent preventive treatment of malaria in pregnancy (IPTp) are contraindicated in the first trimester of pregnancy, when many women are already infected and when rapid diagnostic tests (RDTs) and microscopy miss many infections [7]. Also, studies reported that there was widespread parasite resistance to the drugs recommended for IPTp [8], and sub-patent infections occurred, causing low birthweight and preterm delivery [9]. Therefore, for the detect and treat low-grade infections, more sensitive diagnostic tests are needed. Ensuring detection of all suspected cases will reduce the overuse of artemisinin-based combination therapy or other anti-malarials to reduce the drug pressure on parasites [1, 10].

As such, the main questions surrounding this issue are (1) whether low density infections that are missed by rapid diagnostic tests (RDTs) and by microscopy during pregnancy have deleterious impacts on the mother and developing fetus, and (2) whether the currently available tests in the market can adequately detect these infections in a field setting, where most pregnant women who are infected with malaria present with illness or for antenatal care and may be screened or tested.

Currently used malaria diagnostic tests such as microscopy and rapid antigen-detecting tests (RDTs) are not reliable in detecting low-density infections [10, 11]. Polymerase chain reaction (PCR) detects parasite DNA, and can identify infections below the threshold of detection for microscopy and RDTs [12]. However, PCR requires sophisticated laboratory infrastructure and advanced training, making it challenging and costly to implement in most malaria-endemic areas, where resources are limited [4].

Loop-mediated isothermal amplification (LAMP) was developed based on specific gene amplification. Similar to PCR, LAMP is a molecular technique that amplifies nucleic acids but uses simpler equipment and is less time intensive [4, 13]. An empirical study revealed that LAMP can provide the results within 60–90 min of starting sample processing when carried out by technicians with no previous training in molecular diagnostic techniques, but only given three days of training on LAMP procedures [4]. In the context of malaria elimination, the development of field-ready assays that can detect infection early enough to enable treatment is, therefore, a major priority for malaria elimination [5].

Moreover, many (if not most) pregnant women with malaria infection remain asymptomatic [14], and it is likely that the limit of detection (LOD) of microscopy and RDTs have the lower detection limit of approximately 50–100 parasites per µL(p/µL) of blood [15], which is much higher than the WHO-recommended limit of ≤ 200 IE/100 µL [16]. For instance, studies reported that the LOD was 3.73 ± 0.33 p/µL for Pan LAMP, 4.15 ± 0.36 p/µL for nested PCR [17], < 1 p/µL for quantitative PCR (QRT-PCR) [18], 100–200 p/µL for conventional rapid diagnostic test (cRDT) of blood in field studies, and 0.1-1.0 p/µL for ultrasensitive (uRDT) [19], and 4–20 p/µL in laboratories with expert microscopists and approximately 200 p/µL in field conditions with inexperienced microscopists [18]. Hence, LAMP has potential for detection of malaria in pregnancy. However, published studies with LAMP were performed differently with variations in sample sizes, parasitaemia densities, gravida, and so on. Hence, it is worthy to conduct a meta-analysis, which uses statistical techniques to combine and compare data from different studies, thus increasing the power of the estimates of diagnostic accuracy in primary research [20].

Overall, the objective of current study was to evaluate the diagnostic accuracy of LAMP for the detection of uncomplicated malaria in pregnancy by meta-analysis of data from eligible studies.

## Methods

This meta-analysis was reported, according to the Preferred Reporting Items for Systematic Reviews and Meta-Analysis for Diagnostic Test Accuracy (PRISMA-DTA) guideline [21]. The completed PRISMA-DTA checklist is presented in Additional File 1. A protocol of this meta-analysis study was approved by the Institutional Joint Committee on Research and Ethics ((ID: BMS I-0.2020(19)), and available in INPLASY registration [22].

### Study search

Relevant studies were searched in health-related electronic databases of PubMed, Ovid, Google Scholar, Cochrane Library, the Latin American and Caribbean Health Sciences Literature (LILACS) and African Journals Online (AJOL). Searches were limited to published studies in English language until December 2021. The search was updated on 16 October 2022. The search was performed using keywords and Boolean operators: *(“malaria” [Title/Abstract] OR “plasmodium” [Title/Abstract]) AND (“LAMP” [Title/Abstract] OR “Loop-mediated isothermal amplification” [Title/Abstract]) AND “Pregnancy” [Title/Abstract] AND “diagnosis” [Title/Abstract].* To capture additional studies, references of potentially relevant studies and systematic reviews were manually checked.

### Study selection

Studies were selected according to the criteria stated below.

### Population

Pregnant women, regardless of age, and parity in malaria endemic areas.

### Index test

Any type of LAMP for diagnosis of malaria.

### Reference test

Currently available reference tests such as microscopy and PCR or any comparator test such as RDT. The reference standard is required to be performed using the same blood samples drawn for the index test.

### Target conditions

Detection of human malaria cases, regardless of parasite species.

## Outcome

The outcome of interest was the diagnostic performance of LAMP test measured with sensitivity and specificity of the index test. Included studies, therefore, must have sufficient data on true positive (TP), true negative (TN), false positive (FP) and, false negative (FN) to create a 2 × 2 table. More details about these indices are provided in Additional File 2.

Sensitivity refers to the probability that the index test result is positive in infected cases. Specificity refers to the probability that the index test result is negative in a non-infected case [23, 24].

## Type of studies

Any study design that evaluated the performance of LAMP in the detection of malaria.

## Exclusion criteria

Studies that did not meet the inclusion criteria were excluded. Moreover, studies that assessed other special group of population such as travellers were excluded. This is because the acquisition of immunity in a non-endemic population is different from the pregnant population in endemic areas.

### Data extraction

Two investigators (JLY, CN) independently screened the titles and abstracts, and then selected full-text articles corresponding to the inclusion criteria. The same two investigators extracted information from each included study using a data extraction form prepared for this meta-analysis. At the study level, information collected from each study included author, publication year, characteristics of study participants (median age, sex, parity), characteristics of study design (study design, sample size, study location/setting, study period), tests used in the study (index test, reference standard, blinding of index test interpretation, blinding for reference test interpretation) and test performances (TP, FP, FN, TN). Any discrepancies throughout these processes were settled by a discussion with the third investigator (WST) and reached a consensus.

### Methodological quality assessment

Two investigators (JLY, CN) separately evaluated the methodological quality of the included studies using a revised tool for the quality assessment of diagnostic accuracy studies (QUADAS-2) tool [25]. This tool encompasses four domains such as “patient selection”, “index test”, “reference standard”, and “flow and timing”. Each domain was given signalling questions to assess the risk of bias (RoB), and the applicability. The response “yes”, “no” or “unclear” was given for each signalling questions, whereas the “low”, “unclear” or “high” was given for the judgement of RoB. Any discrepancies between the two investigators were also settled by a discussion with the third investigator (NHH/HHA) and reached a consensus.

### Data synthesis

Test performance indicators used in this study were sensitivity and specificity along with their 95% confidence intervals (CIs). The details for these indicators are presented in Additional File 2. If minimum of four studies were eligible, a pooled analysis was performed. Hence, pooled analyses were available for all studies regardless of reference tests, and for studies that used miscopy or PCR as reference tests. For pooling of studies, sensitivity and specificity including 95% CI of each study were combined using a random effects model, and were illustrated with a forest plot. *I*^*2*^ statistic determines the total percentage of variation across studies that is attributable to heterogeneity instead of chance. A value of 0% suggests no observed heterogeneity, while a value > 50% is considered substantial heterogeneity [26].

For the overall test performance across different threshold, summary receiver operating characteristics (SROC) plots were created, following the methods described elsewhere [27], An important indicator from SROC curves is the area under the curve (AUC) which serves as a global measure for test performance. As described elsewhere [28], the AUC values between 0.5 and 0.7, 0.7 to 0.9 and 0.9 to 0.9 represent low, moderate and high accuracies, respectively.

Meta-regression analysis was conducted to investigate sources of heterogeneity between studies. Different covariates were used such as sample size, risk of bias, study design, reference test, blind test and blind reference. A p-value < 0.1 was used to indicate significant heterogeneity. Publication bias was not done as this test was not recommended for DTA studies [27, 29]. For data analysis, *midas* package of STATA (STATA Txt 16) was used.

## Results

**Fig. 1:**
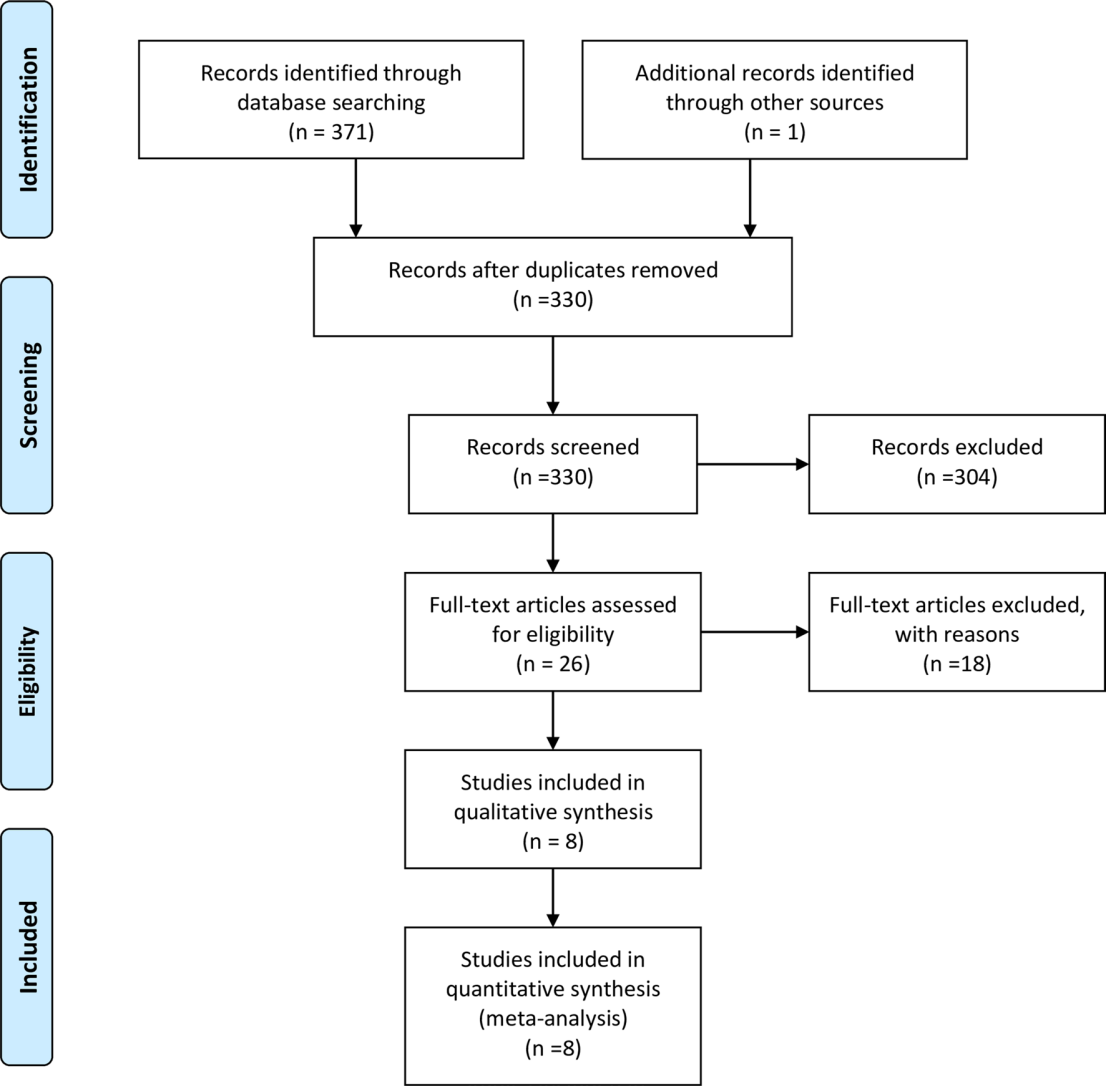
Study selection process

Figure 1. shows the PRISMA flow diagram of the study selection process. Initial searches yielded 372 studies. Then, 42 duplicates and 304 irrelevant articles were removed after title and abstract screen, and 26 full-text articles were assessed. A final eight studies [30–37] were eligible in this review. The reasons for the exclusion of 18 studies were listed in Additional file 3.

### Characteristics of the included studies

Table 1 summarizes the main characteristics of the eight studies (9 datasets) identified. These studies encompassed 2999 participants, ranging from a minimum 87 [33] to maximum 858 [36]. The median age of participants varied from 21 years [30] to 26.2 years [32]. In this review, pregnant women in two studies that compared IPTp every eight-week sulfadoxine-pyrimethamine (SP) to four-week or eight-week regimens of dihydroartemisin-piperaquine (DP) [30], and daily trimethoprim-sulfamethoxazole (TMP-SMX) plus monthly DP to daily TMP-SMX alone [31]. The majority of participants used DP (i.e. more than 51.5%) [30, 34]. The studies included were published between 2014 and 2022. Two studies were done in Colombia [36, 37], three studies in Uganda [32–34], and one study each in Kenya [37], Northwest Ethiopia [33], Southwest Ethiopia [32]. Two studies each were randomized controlled trials [31, 34], cross-sectional design [31, 36], or prospective study [35, 37]. and one each study was a longitudinal study [32], or nested cohort [30]. Three studies used microscopy [30, 31, 34], one study used microscopy as well as RDT (HRP2/pLDH combo) [32] or PCR as well as cRDT and uRDT [37], and the remaining three studies used PCR as reference tests [33, 35, 36]. All of these studies used Pan-LAMP, and one study also used *Pf*LAMP to retest samples that had previously tested positive for Pan-LAMP [35].

**Table 1:** Characteristics of the studies included in the meta-analysis

Author, yr	Study yr	Mean (SD) age in yr	Gravida	Study design	Country	Index test	Manufacture/brand name	Ref test	tp	fp	fn	tn	Sample size	Parasite density	Remarks
Kapisi 2017 [30]	2014	21(4.2)^$^	Pri (46.6%)^$^	Nested cohort	Uganda	LAMP	Eiken Loopamp™	Microscopy	24	0	1	255	280	NA	HIV -ves
Natureeba 2017 [31]	2014	30	Multi (82%)	RCT	Uganda	LAMP	Eiken, Japan	Microscopy	0	0	2	96	98	NA	HIV + ves
Tadesse 2020 [32]	2018–2019	26.2 (4.6)	Multi (75.8%)	L	Southwest Ethiopia	LAMP	Meridien Bioscience, Cincinnati, OH	Microscopy	5	4	0	426	435	mean 882.7 p/µL	
	2018–2019	26	Multi (56.0%)	L	Southwest Ethiopia	LAMP	Meridien Bioscience, Cincinnati, OH	RDT (HRP2/pLDH combo)	6	3	0	426	435		
Tegegne 2017 [33]	2016	27	Multi (74.7%)	CS	Northwest Ethiopia	LAMP (Pan/Pf)	Eiken Chemicals, Tokyo	nPCR	10	5	0	72	87	3380 p/µL	
Tran 2020 [34]	2014	21(md)	Pri (38.2%)	Secondary data from an RCT	Uganda	LAMP	Eiken Chemical	Microscopy	23	0	78	127	228	NA	
Vasquez 2018 [35]	2016–2017	24 (IQR, 20–28)^1^	Pri (49.3%)	Prospective	Colombia	Pan-LAMP, Pf-LAMP	Eiken Chemicals, Tokyo	nPCR	39	0	0	492	531	2480 p/µL(md)	
Vasquez 2020 [36]	2017–2018	24 (IQR, 20–29)^1^	Pri (30.5%)	CS	Colombia	Pan-LAMP, Pf-LAMP	Loopamp™ MALA RIA Kit, Eiken Chemical Company	qRT-PCR	35	1	4	818	858	geometric mean of 13.2 p/µL	
Samuels 2022 [37]	2018	23 (IQR 20–28)	Pri (25.5%) Section (25.9%) multi (48.6%)	Prospective	Kenya	LAMP	Meridian Bioscience, Illumigene Malaria	PET PCR	118	55	54	310	482	< 200 p/µL^$$^	

*LAMP*: loop-mediated isothermal amplification; multi: multiparous; md: medium; *nPCR*: nested polymerase chain reaction; *pLDH*: Parasite lactate dehydrogenase; *Pan-LAMP*: LAMP for all *Plasmodium* species; *Pf-LAMP* LAMP specific for *Plasmodium falciparum*; *pri* primary gravida; *qRT-PCR* Real-Time Quantitative Reverse Transcription polymerase chain reaction; *RCT* Randomized controlled trial; *RDT*: Rapid diagnostic test; Ref test: reference test; sec: secondary; *SP* Sulfadoxine–Pyrimethamine ; *TN* true negative; *TP* true positive; *yr* year *FN* false negative; *FP* false positive;* HRP2* Histidine rich protein 2; *IQR* interquartile range; *L* longitudinal

^$^: ≥ 50% has positives; $$: 78.5% of CS: cross-sectional

### Methodological quality of included studies

Methodological quality of each study identified for the present meta-analysis and summary of the methodological quality of studies are presented in Additional File 4. Overall, none of these studies posed a low RoB. It was unclear whether the index test was evaluated blindly or the detection power of the reference test contributed to this. Regarding “the applicability” assessment, almost all were with low concerns, based on “patient selection”, “index test” and “reference standard”.

### Test performance

**Fig. 2:**
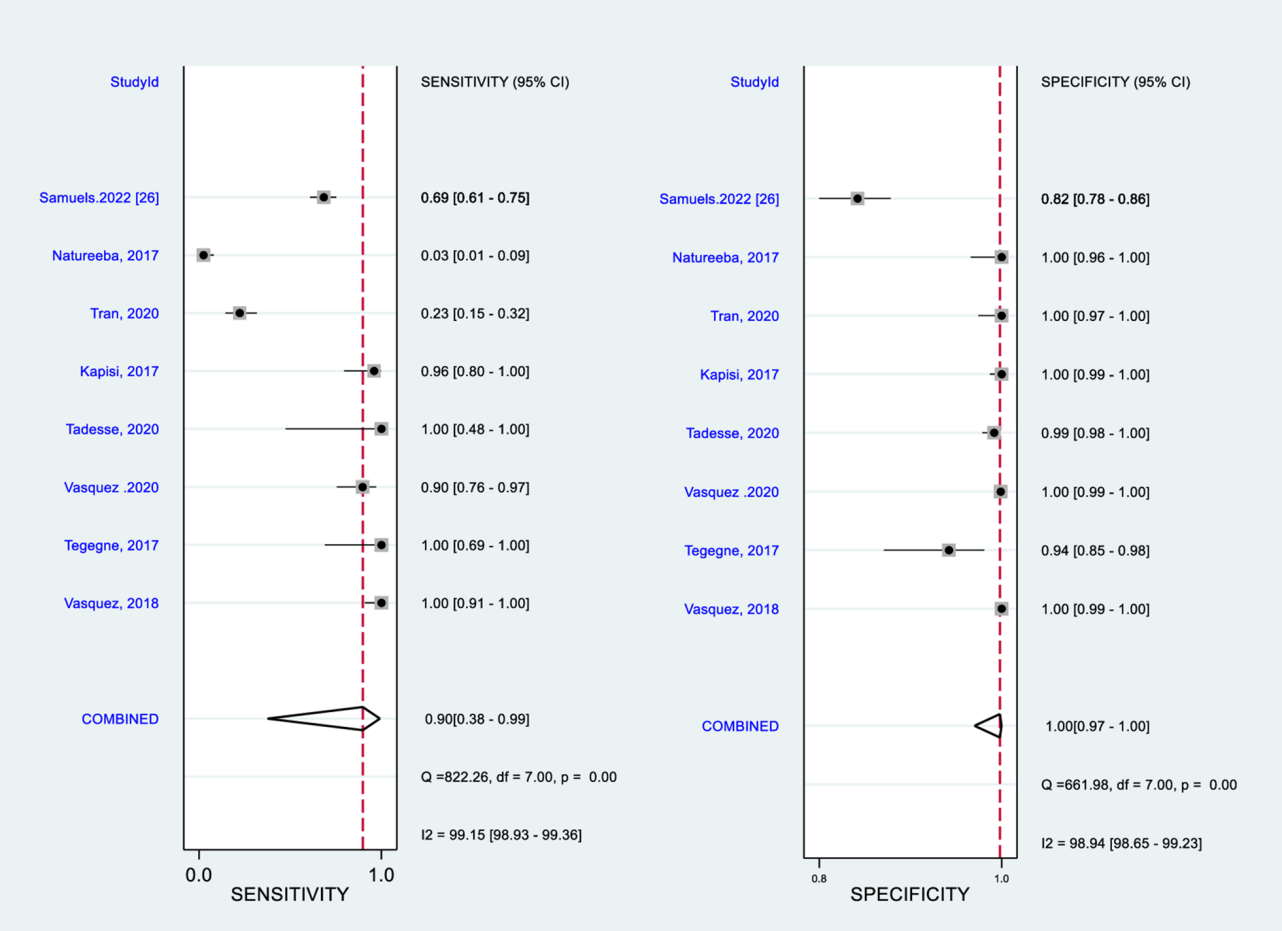
Pooled diagnostic accuracy of LAMP using any reference tests for detection of malaria in pregnancy

The pooled sensitivity and specificity of LAMP (Pan LAMP) for detection of malaria in pregnant women, regardless of any reference tests are 0.90 (95%CI 0.38–0.99, 8 studies, *I*^*2*^ test:99%) and 1.00 (95%CI 0.97-1.00, 8 studies, *I*^*2*^ test: 98.9%), respectively (Fig. 2). This showed that the ability of LAMP (Pan LAMP) test to accurately identify a person as ′infected′ (presence of malaria) ranged widely from 30 to 100%. The ability of LAMP test to accurately identify a person as ‘not infected’ (absence of malaria) ranged from 99 to 100%. However, there was substantial between-study heterogeneity (*I*^*2*^ test > 98% in both indices), and interpretation should be with great caution.

**Fig. 3:**
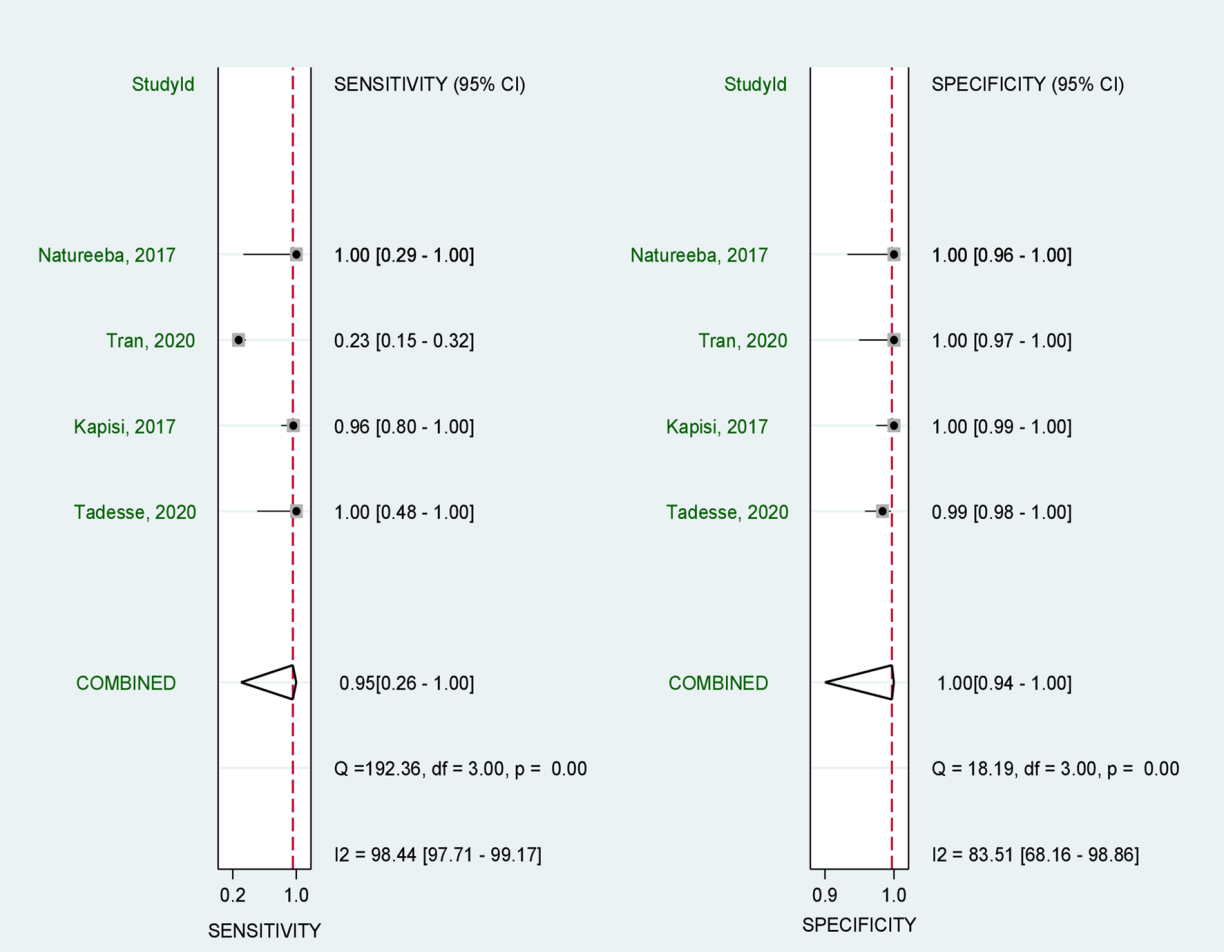
Pooled diagnostic accuracy of LAMP using microscopy as reference test for detection of malaria in pregnancy

The pooled sensitivity and specificity of LAMP (Pan LAMP) for detection of malaria in pregnant women, using microscopy as a reference test was 95% (95%CI 26–100%, 4 studies, *I*^*2*^ test: 98.4%) and 100% (95%CI 94–100%, 4 studies, *I*^*2*^ test: 83.5%), respectively (Fig. 3). Using microscopy as a reference test, the ability of this test to accurately identify a person as ′infected′ (presence of malaria) widely ranged from 26 to 100%. The ability of this test to accurately identify a person as ‘not infected’ (absence of malaria) ranged from 94 to 100%. However, this was with substantial between-study variations (*I*^2^ test: >83% in both indices). Hence, interpretation should be with great caution.

The SROC curve for studies using any reference test (Additional File 5), or microscopy (Additional File 6) as a reference test indicates the AUC (i.e., the global measure for test performance) is 100% (95%CI 99–100%). This implied a high accuracy of the diagnostic performance of LAMP (Pan LAMP). The more these values are, the more capacity to detect the presence of malaria parasite. Due to the limited number of studies, there is concern over the power of this estimation.

Table 2 presents the diagnostic performance of individual studies that used PCR as reference test. Four studies were identified for this comparison (Additional File 7). Overall, pooled sensitivity of LAMP compared to PCR is 91% (95%CI 67–98%), while specificity is 99% (95%CI 83–100%). Regarding different type of PCR (particularly their respective target DNA or total nucleic acid) with different performance, interstudy variation was also reported (Table 2). Interstudy variation in sensitivity compared with PCR is 37% (95% CI 4–69%), while it is 79% (95% CI 73–85%) for specificity. This mean the pooled specificity is less confidence than the pooled sensitivity, and this could be accounted for an interpretation. Moreover, the negative likelihood ratio was 9% (2–40%). Thus, cautious interpretation is needed due to the substantial between-study heterogeneity (*I*^2^ test: 80%), and a low probability that a person without infection is stated negative. It is important to note that three different types of PCR were used in these investigations, and each type’s performance varied.

**Table 2:** Diagnostic accuracy of LAMP with PCR reference test

Author, yr [ref no.]	TP	FP	FN	TN	Sample size	PCR type	Respective target	Sensitivity	Specificity
Tegegne, 2017 [33]	10	5	0	72	87	nPCR	Small subunit RNA (ssrRNA) genes	1.00 [0.69, 1.00]	0.94 [0.85, 0.98]
Vasquez, 2018 [35]	39	0	0	492	531	nPCR	Small subunit RNA (ssrRNA) genes	1.00 [0.91, 1.00]	1.00 [0.99, 1.00]
Vasquez 0.2020 [36]	35	1	4	818	858	qRT-PCR	18 S rRNA genes	0.90 [0.76, 0.97]	1.00 [0.99, 1.00]
Samuels.2022 [37]	118	55	54	255	482	PET-PCR	18 S ssrRNA genes	0.69 (0.61–0.75)	0.82 (0.77–0.86)
Pooled								0.91(0.67-98)	0.99(0.83–100)
*I*^2^test								99.4%	99.6%

Negative Likelihood Ratio=0.09 (0.02–0.4)

*FN* false negative; *FP* false positive; *nPCR* nested polymerase chain reaction; *PET-PCR* photoinduced electron transfer polymerase chain reaction; *qRT-PCR* Real-Time Quantitative Reverse Transcription polymerase chain reaction; *Ref* reference; *TN* true negative; *TP*: true positive; *yr*: year

For investigating the source of heterogeneity, a meta-regression analysis was performed with three covariates such as study design, sample size, reference tests (including different types of PCR) and blindness of test. Since there was insufficient information available for parasitaemia, and different types of LAMP, they were not included as covariates. Of these potential confounding factors, reference test (p: 0.03) and study design (p: 0.03) had affected the diagnostic accuracy of LAMP in malaria in pregnancy (Additional File 8).

## Discussion

The present meta-analysis included seven studies with 2999 participants from three endemic countries. Below is the summary of findings.

1. All studies included were from the endemic countries of Africa and Americas.
2. Some of the microscopy negative cases were classified as positive by LAMP when compared to reference test microscopy.
3. The nature of study design and type of reference tests had an impact on the sources of between- study heterogeneity.
4. Overall, there is insufficient evidence in diagnostic performance of LAMP for detecting malaria in pregnant women.

The majority of the studies were carried out in malaria endemic populations. The WHO African region had about 94% of global malaria cases, whereas the WHO Region of the Americas accounted for 0.39% of global malaria cases [38]. Hence, the current findings including only one study that conducted in Africa did not reflect the geographic distribution of endemic population relevant to the African context.

The importance of timing of infection on the development of placental malaria may vary with the gravida of pregnancy. As such, burden of infection and timing of parasitaemia impacted on the risk for placental malaria [34]. This could contribute to a large variation in the sensitivity of LAMP observed in the current analysis, where both multigravida and primigravid have been included. Transmission intensity of malaria could have impact on variation in the sensitivity of LAMP observed in this current analysis. In high transmission areas, women have acquired immunity during their life, and although they might have substantial placental sequestration (especially in primi- and secundigravidae), they can have a lower number of parasites in circulation. In low transmission areas, women have low or no immunity and women can get sick at initially low parasite densities [36]. Due to paucity of these data, it was not possible to stratify the diagnostic accuracy of LAMP either by gravida, parasite densities, or transmission intensity.

There were discrepancies between the LAMP test and the reference tests. This highlights the need for more sensitive PCR technique to accurately evaluate the performance of LAMP [33]. Studies reported difference in detection infections between the methods used in malaria in pregnancy. For instance, a systematic review incorporating studies solely in Columbia documented that prevalence of malaria in pregnancy by microscopy was 4.5% (95%CI 2.9–6.9%), while this was 14.4% (95%CI 7.6–25.5%) with PCR, indicating higher diagnostic yield of PCR to detect cases in pregnant women [39]. It is important note that acquired immunity may partially control malaria infection and hence increase the possibility of sub-microscopic parasitaemia during malaria in pregnancy [32, 40] and harbour low level infections [33]. This might have an effect of low detection power of malaria in pregnancy, especially when microscopy is used as a reference test in asymptomatic or low-density infections. Moreover, IPT with DP could be associated with a lower burden of malaria as compared to the IPT with SP among HIV-uninfected pregnant women [31]. Only 2 of the seven studies included in this review provided information on participants with IPT [30, 34]. Number of pregnant women with IPT treatments with DP was proportionally more than that of SP in both studies included. This might have confounded actual diagnostic accuracy of malaria in pregnant women.

If malaria is not detected in the first trimester, existing undernutrition may worsen the already increased susceptibility to malarial infection, impair development of protective immunity to malaria, and is likely to exacerbate the impact of placental malaria on fetal growth [41]. Placental malaria causes local inflammatory cytokine and chemokine generation, which is associated with low birth weight, pre-mature birth and foetal growth restriction [41, 42].

### Implications

Pregnant women are more susceptible to malaria, have low level infections, and suffer from malaria related complications themselves and new-borns [40]. Hence, early detection of malaria with a highly sensitive, field-friendly detection method in the context of an antenatal care program is crucially important. Current commercially available LAMP technology devices and reagents are expensive, limiting their use in low and middle-income countries. LAMP technology could help detect malaria in pregnant women through screening strategies, but only if affordable LAMP test is made available [40].

Furthermore, the current findings suggest that LAMP has limited accuracy in detecting malaria in pregnant women. According to the published reviews, both undiagnosed submicroscopic infections and asymptomatics are a barrier to the control and elimination programs by allowing the parasite to permanence of parasite reservoirs and thereby determining the intensity and stability of malaria transmission, especially in low endemic areas [39, 43].

Antenatal malaria detection with a highly sensitive, affordable method that can detect infections in the first trimester and tailored management of high-risk mothers may help to prevent adverse pregnancy outcomes caused by malaria, and other risk factors (e.g. malnutrition). As such, potential target groups include primigravid, undernourished, HIV positives, or anaemic women in particular who may benefit from improved malaria prevention strategies combined with appropriate nutritional supplementation, delivered conjointly through antenatal care systems [41]. LAMP is simpler to implement than other more complex molecular methods. However, a number of sample processing steps are needed, and the higher cost-per-test compared to RDTs should be addressed [37].

#### Study limitations

Among the included studies, the types of samples that were assessed were not all the same. For example, one study matched placental histopathology, which could detect prior placental malaria and found a low sensitivity of 0.23 [30]. This might be due to a fact that if these women were treated during pregnancy, they might have cleared the infection by the time of delivery, thus the histology negative results. With early testing in the first trimester, sequestration leads to low peripheral parasitaemia that typically occurs after placenta formation is complete, which explains the lower sensitivity of microscopy. At the histological level, the sequestration does not explain the negative microscopy result. It can be low parasitaemia or submicroscopic placental parasitaemia [34, 4]. Two small studies (i.e. less than 100 sample size) identified for this review [31, 33] could have contributed to the low statistical power. There is also inherent limitation to the LAMP. Although this meta-analysis documented that the diagnostic accuracy of LAMP in pregnant women is high, the overall quality of evidence is low owing to the small number of studies, different in study design and reference tests as well as the risk of bias in methodological quality of the included studies. Hence, the accuracy estimates reported in this study should be interpreted with great caution.

## Conclusion

The findings suggest that LAMP is more sensitive than traditional tests used at facilities (microscopy and RDTs), but the utility of detecting and treating these low-density infections is not well understood. Due to the limited number of studies with bias in their methodological quality, and variation in the study design, further research is likely to change the estimate. Well-conceived prospective studies with larger samples and blinding of the index test results are recommenced.

## Supplementary Information

**AdditionalFile 1: Table S1.** PRISMA-DTA Checklist.

**AdditionalFile 2:** Descriptions of indices.

**Additional File 3: Table S2.** Summary of excluded studies.

**AdditionalFile 4: Figure S1.** Summary of the methodological quality assessment.

**AdditionalFile 5: Figure S2.** SROC curve for studies using any reference test.

**AdditionalFile 6: Figure S3.** SROC curve for studies using microscopy as reference test.

**AdditionalFile 7: Figure S4.** Pooled diagnostic accuracy of LAMP using PCR as reference test.

**AdditionalFile 8: **Meta-regression indicating covariates.

## Data Availability

All data generated or analysed during this study are included in this article and its Additional files.

## Abbreviations

AUC: Area under the curve
AJOL: African journals online
CI: Confidence interval
cRDT: Conventional rapid diagnostic test
DP: Dihydroartemisin-piperaquine
DTA: Diagnostic test accuracy
GTS: Global technical strategy
LAMP: Loop-mediated isothermal amplification
LILACS: The Latin American and Caribbean health sciences literature
nPCR: Nested polymerase chain reaction;
PRISMA-DTA: Preferred reporting items for systematic reviews and meta-analysis for diagnostic test accuracy
RDT: Rapid-onsite diagnostic test
qRT-PCR: Real-time quantitative reverse transcription polymerase chain reaction
QUADAS-2: A revised tool for the quality assessment of diagnostic accuracy studies
SP: Sulfadoxine-pyrimethamine
TMX: Trimethoprim-sulfamethoxazole
uRDT: Ultrasensitive rapid diagnostic test
