# Supplementary material for: Loop-mediated isothermal amplification (LAMP) test in the detection of uncomplicated malaria in pregnancy: a meta-analysis of diagnostic accuracy
Source: Malar J. 2022 Dec 22;21:391. doi: 10.1186/s12936-022-04419-9 (PMC9783437; doi:10.1186/s12936-022-04419-9)
Supplement: Supplementary file 2 — AdditionalFile 2: Descriptions of indices. [file 12936_2022_4419_MOESM2_ESM.doc]

**Additional File** **2 Descriptions of indices**

**True positive (TP), true negative (TN),** **false positive (FP) and,** **false negative (FN)**

“TP” refers to the number of people that the index test correctly identified as having the condition (i.e. the number of participants that tested positive with both the index test and reference standard). “FP” refers to the number of people that the index test incorrectly identified as having the condition (i.e. the number of people that tested positive with the index test but negative with the reference standard). “TN” refers to the number of people that the index test correctly identified as not having the condition (a negative result with both the index and the reference standard test). “FN” refers to the number of people that the index test incorrectly identified as not having the condition (a negative result on the index test but positive with the reference standard).

Source: WHO Handbook for Guideline Development, 2^nd^ Edition. 17. Developing guideline recommendations for tests or diagnostic tools

<https://www.who.int/publications/guidelines/Handbook_for_guideline_development-Chapter_17-Diagnostic_tests.pdf?ua=1>

**Sensitivity and specificity of the test**

The sensitivity of a test is defined as the probability that the index test result will be positive in a diseased case. Sensitivity is sometimes referred to as Detection Rate (DR), True Positive Rate (TPR) or True Positive Fraction (TPF).  It is expressed either as a proportion or a percentage.

The specificity of a test is defined as the probability that the index test result will be negative in a non‐diseased case.  Specificity is occasionally referred to as the True Negative Rate (TNR) or True Negative Fraction (TNF).  More often, the terms False Positive Rate (FPR) and False Positive Fraction (FPF) are used for the complement of specificity (computed as 1−specificity or b/(b+d)).  Again, both proportions and percentages are used.

**The positive likelihood ratio** describes how many times more likely positive index test results were in the diseased group compared to the non‐diseased group. The positive likelihood ratio, which should be greater than 1 if the test informative, is defined as:

LR+ = P(T+|D+)/P(T+|D‐) = sens/(1−spec), and is estimated as (a/(a+c)) / (b/(b+d)).

**The negative likelihood ratio** describes how many times less likely negative index test results were in the diseased group compared to the non‐diseased group. The negative likelihood ratio, which should be less than 1 if the test is informative,  is defined as:

LR‐ = P(T‐|D+)/P(T‐|D‐) =   (1–sens)/spec, and is estimated as (c/(a+c)) / (d/(b+d)).

**Source:**

Macaskill P, Gatsonis C, Deeks JJ, Harbord RM, Takwoingi Y. Analysing and presenting results. In: Deeks JJ, Bossuyt PM, Gatsonis C (Eds), Cochrane Handbook for Systematic Reviews of Diagnostic Test Accuracy Version. Chapt 10. London. The Cochrane Collaboration, 2013. Available from: <http://srdta.cochrane.org/>
