# Supplementary material for: Loop-mediated isothermal amplification (LAMP) test in the detection of uncomplicated malaria in pregnancy: a meta-analysis of diagnostic accuracy
Source: Malar J. 2022 Dec 22;21:391. doi: 10.1186/s12936-022-04419-9 (PMC9783437; doi:10.1186/s12936-022-04419-9)
Supplement: Supplementary file 3 — Additional File 3: Table S2. Summary of excluded studies. [file 12936_2022_4419_MOESM3_ESM.doc]

Additional File 3. Table S2. Summary of excluded studies

| No | Study | Reasons | Reference citation |
| --- | --- | --- | --- |
| 1 | Conrad, 2017 | Insufficient data to construct 2x2 diagnostic table | Conrad MD, Mota D, Foster M, et al. Impact of intermittent preventive treatment during pregnancy on Plasmodium falciparum drug resistance–mediating polymorphisms in Uganda. J Infect Dis 2017;216(8):1008-1017 |
| 2 | Chen, 2021 | Population was not pregnant women | Chen X, Pan M, Huang Y, Qin P, et al. Detection of Falciparum Malaria Imported From Africa With a Highly Specific and Sensitive Loop-Mediated Isothermal Amplification (LAMP) Assay. 2021 |
| 3 | Hartmeyer, 2019 | Population was not pregnant women | Hartmeyer GN, Hoegh SV, Skov MN, et al. Use of loop-mediated isothermal amplification in a resource-saving strategy for primary malaria screening in a non-endemic setting. Am J Trop Med Hyg 2019;100(3):566-571. |
| 4 | McCreesh, 2018 | Population was not pregnant women | McCreesh P, Mumbengegwi D, Roberts K, et al. Subpatent malaria in a low transmission African setting: a cross-sectional study using rapid diagnostic testing (RDT) and loop-mediated isothermal amplification (LAMP) from Zambezi region, Namibia. Malaria journal 2018;17(1):1-11. |
| 5 | Mukhtar, 2018 | The diagnosis of malaria was not investigated | Mukhtar M, Ali SS, Boshara SA, et al. Sensitive and less invasive confirmatory diagnosis of visceral leishmaniasis in Sudan using loop-mediated isothermal amplification (LAMP). PLoS neglected tropical diseases 2018;12(2):e0006264 |
| 6 | Katra, 2017 | Population was not pregnant women | Katrak S, Murphy M, Nayebare P, et al. Performance of loop-mediated isothermal amplification for the identification of submicroscopic Plasmodium falciparum infection in Uganda. Am J Trop Med Hyg 2017;97(6):1777-1781. |
| 7 | Prahl, 2016 | Insufficient data to construct 2x2 diagnostic table | Prahl M, Jagannathan P, McIntyre TI, Auma A, Farrington L, Wamala S, et al. Timing of in utero malaria exposure influences fetal CD4 T cell regulatory versus effector differentiation. Malaria journal 2016;15(1):1-10. |
| 8 | Conroy , 2019 | Insufficient data to construct 2x2 diagnostic table | Conroy AL, Bangirana P, Muhindo MK, et al. Case report: birth outcome and neurodevelopment in placental malaria discordant twins. Am J Trop Med Hyg 2019;100(3):552-555. |
| 9 | Conroy , 2012 | Insufficient data to construct 2x2 diagnostic table | Conroy AL, McDonald CR, Kain KC. Malaria in pregnancy: diagnosing infection and identifying fetal risk. Expert review of anti-infective therapy 2012;10(11):1331-1342. |
| 10 | Sama, 2015 | Population was not pregnant women | Sema M, Alemu A, Bayih AG, et al. Evaluation of non-instrumented nucleic acid amplification by loop-mediated isothermal amplification (NINA-LAMP) for the diagnosis of malaria in Northwest Ethiopia. Malaria journal 2015;14(1):1-9. |
| 11 | Lucchi, 2018 | Insufficient data to construct 2x2 diagnostic table | Lucchi NW, Ndiaye D, Britton S, et al. Expanding the malaria molecular diagnostic options: opportunities and challenges for loop-mediated isothermal amplification tests for malaria control and elimination. Expert review of molecular diagnostics 2018;18(2):195-203. |
| 12 | Eun, 2013 | Population was not pregnant women | Han E. Loop-mediated isothermal amplification test for the molecular diagnosis of malaria. Expert review of molecular diagnostics 2013;13(2):205-218. |
| 13 | Hopkins, 2013 | Population was not pregnant women | Hopkins H, González IJ, Polley SD, et al. Highly sensitive detection of malaria parasitemia in a malaria-endemic setting: performance of a new loop-mediated isothermal amplification kit in a remote clinic in Uganda. J Infect Dis 2013;208(4):645-652. |
| 14 | Kajubi, 2019 | Insufficient data to construct 2x2 diagnostic table | Kajubi R, Ochieng T, Kakuru A, et al. Monthly sulfadoxine–pyrimethamine versus dihydroartemisinin–piperaquine for intermittent preventive treatment of malaria in pregnancy: a double-blind, randomised, controlled, superiority trial. The Lancet 2019;393(10179):1428-1439. |
| 15 | Ategeka, 2020 | Insufficient data to construct 2x2 diagnostic table | Ategeka J, Kakuru A, Kajubi R, et al. Relationships between measures of malaria at delivery and adverse birth outcomes in a high-transmission area of Uganda. J Infect Dis 2020;222(5):863-870. |
| 16 | Unwin, 2020 | Insufficient data to construct 2x2 diagnostic table | Unwin VT, Ahmed R, Noviyanti R, et al. Use of a highly-sensitive rapid diagnostic test to screen for malaria in pregnancy in Indonesia. Malaria journal 2020;19(1):1-8. |
| 17 | Kakuru,2016 | The same population of an included study | Kakuru A, Jagannathan P, Muhindo MK, et al. Dihydroartemisinin–piperaquine for the prevention of malaria in pregnancy. N Engl J Med 2016;374(10):928-939. |
