# Supplementary figures and images for: Loop-mediated isothermal amplification (LAMP) test in the detection of uncomplicated malaria in pregnancy: a meta-analysis of diagnostic accuracy
Source: Malar J. 2022 Dec 22;21:391. doi: 10.1186/s12936-022-04419-9 (PMC9783437; doi:10.1186/s12936-022-04419-9)

**Additional File 4: Figure S1.** **Summary of the methodological quality assessment**


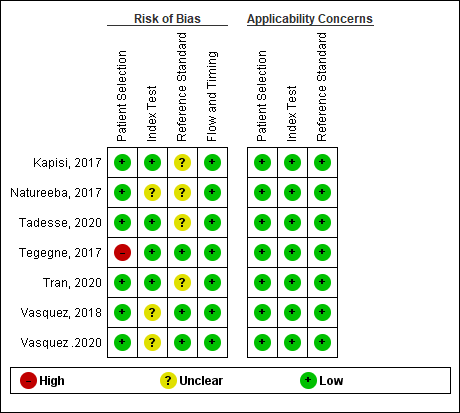


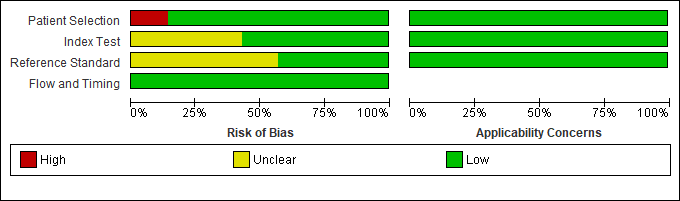

Supplement: Supplementary file 4 — AdditionalFile 4: Figure S1. Summary of the methodological quality assessment. [file 12936_2022_4419_MOESM4_ESM.doc]
