# Supplementary material for: Loop-mediated isothermal amplification (LAMP) test in the detection of uncomplicated malaria in pregnancy: a meta-analysis of diagnostic accuracy
Source: Malar J. 2022 Dec 22;21:391. doi: 10.1186/s12936-022-04419-9 (PMC9783437; doi:10.1186/s12936-022-04419-9)
Supplement: Supplementary file 5 — AdditionalFile 5: Figure S2. SROC curve for studies using any reference test. [file 12936_2022_4419_MOESM5_ESM.doc]

**Additional File 5: Figure S2. SROC curve for studies using any reference test**
