# Supplementary material for: Loop-mediated isothermal amplification (LAMP) test in the detection of uncomplicated malaria in pregnancy: a meta-analysis of diagnostic accuracy
Source: Malar J. 2022 Dec 22;21:391. doi: 10.1186/s12936-022-04419-9 (PMC9783437; doi:10.1186/s12936-022-04419-9)
Supplement: Supplementary file 8 — AdditionalFile 8: Meta-regression indicating covariates. [file 12936_2022_4419_MOESM8_ESM.doc]

**Additional File 8: Meta-regression indicating covariates**

Parameter category LRTChi2 Pvalue I2 I2lo I2hi

+------------------------------------------------------------+

| Parameter category LRTChi2 Pvalue I2 I2lo I2hi |

|------------------------------------------------------------|

| sample 0.63 0.73 0 0 100 |

| reftest 6.91 0.03 71 36 100 |

| blindtest 0.79 0.67 0 0 100 |

| design 7.07 0.03 72 37 100 |

+------------------------------------------------------------+

+--------------------------------------------------------------+
